# Substantiation of Epitaxial Growth of Diamond Crystals on the Surface of Carbide Fe_3_AlC_0.66_ Phase Nanoparticles

**DOI:** 10.1186/s11671-017-1869-3

**Published:** 2017-03-09

**Authors:** Ievgenij M. Dzevin, Alexander A. Mekhed

**Affiliations:** 0000 0004 0482 7152 grid.435300.1 G.V. Kurdyumov Institute for Metal Physics NAS of Ukraine, Vernadsky blvd. 36, Kiev, 03680 Ukraine

**Keywords:** Fe–Al–C alloys, K-phase, Diamond, Epitaxial growth, Electronic structure

## Abstract

Samples of Fe–Al–C alloys of varying composition were synthesized under high pressures and temperatures. From X-ray analysis data, only K-phase with usual for it average parameter of elemental lattice cell, *a* = 0.376 nm, carbide Fe_3_C and cubic diamond reflexes were present before and after cooling to the temperature of liquid nitrogen.

Calculations were made of the parameters of unit cells, the enthalpy of formation of the Fe_3_AlC, Fe_3.125_Al_0.825_C_0.5_, Fe_3.5_Al_0.5_C_0.5_, Fe_3.5_Al_0.5_C, Fe_3_Al_0.66_C_0.66_, and Fe_3_AlC_0.66_ unit cells and crystallographic planes were identified on which epitaxial growth of the diamond phase was possible, using density functional theory as implemented in the WIEN2k package.

The possibility of epitaxial growth of diamond crystals on Fe_3_AlC_0.66_ (K-phase) nanoparticles was, therefore, demonstrated. The [200] plane was established to be the most suitable plane for diamond growth, having four carbon atoms arranged in a square and a central vacancy which can be occupied by carbon during thermal-and-pressure treatment. Distances between carbon atoms in the [200] plane differ by only 5% from distances between the carbon atoms of a diamond. The electronic structure and energetic parameters of the substrate were also investigated. It was shown that the substrate with at least four intermediate layers of K-phase exhibits signs of stability such as negative enthalpy of formation and the Fermi level falling to minimum densities of states.

## Background

The wide range of properties of Fe–Al–C alloys is defined by features of their structural-and-phase state, relative to other alloys of the Fe–Cr/V/Ni/Al/Ti–C systems [1–3], that forms during thermal and mechanical treatment and during the actions of other external factors. To specify the features of Fe–Al–C alloys it is necessary to note the solubility of carbon in the austenitic phase, the abnormally high tetrahonality of the martensitic phase containing nanoscale particles of the carbide phase, the coherency of this carbide phase with the austenitic matrix, which causes abnormally high tetrahonality of martensite, and increased resistance to disintegration of the carbon α-solid solution.

The fundamental investigation of the structural-and-phase state of Fe–Al–C alloys centers around the conditions for the formation and structure of the specific carbide phase, Fe_4–y_Al_y_C_x_, which has a face-centered cubic (f.c.c.) lattice (famously known as K-phase), and to studying its effect on physical and mechanical properties of alloys [4]. This phase has also been referred to as: *ε-*phase, double carbide, K-phase, antiperovskite, metal-like carbide, etc. [5–7], but at the present time the most widely accepted term is K-phase. Indeed, all the published triple-state diagrams of Fe–Al–C alloys refer to K-phase as an Fe_3_AlC composition. As is widely known [8], on every three elemental cells of the f.c.c. K-phase lattice there are only two carbon atoms introduced into the octahedral interstitions and one octahedral interstition remains vacant. Therefore, the Fe_3_AlC formula as being K-phase is not strictly accurate and this inaccuracy is important.

It is assumed that if the powder mixture, which corresponds to stoichiometric Fe_3_AlC composition, under extreme conditions undergoes thermal-and-pressure treatment simultaneously, and quickly cools, it may be possible to fix the K-phase of stoichiometric Fe_3_AlC. However, if this does not succeed and a quantity of carbon atoms is fixed into the tetrahedral or other pores of the K-phase crystal lattice, we can expect some changes in properties of K-phase. In trying to solve this particular problem – to achieve the synthesis of the Fe_3_AlC K-phase – it is necessary to investigate this problem further and also to investigate the alloys with lower contents of aluminum and carbon, and also those with a higher content of carbon: Fe_4–у_Al_у_C_x_ + C.

## Methods

The materials used initially were the powder mixture: ARMCO-iron (99.99 wt. %), chemically pure aluminum, and graphite in the proportions shown in Table 1.

**Table 1:** Contents of the Fe–Al–C system alloys and the regimes of obtained specimens

Number	Contents, wt. %			Regimes		
	Fe	Al	C	P, GPa	T, K	τ, s
1	94	4	2	7.7	1998	60
2	89.5	8	2,5	7	2123	60
3	90.3	5.8	3.9	7.2	1873	60
4	81	13.6	5.4	7.7	2173	60
5	81.1	13.1	5.8	6	1673	60

*Al* aluminum, *Fe* ARMCO-iron, *C* graphite

For thermal-and-pressure synthesis of the Fe–Al–C samples the toroid-type high-pressure apparatus with a hole diameter of 20 mm was chosen. This apparatus can generate a pressure in the reaction volume of more than 8 GPa [9, 10].

The required X-ray investigations were carried out on a DRON-3 X-ray diffractometer using monochromatic Co-Kα irradiation, and images of the fractures, surfaces, and spectral data were obtained on Jeol JSM 6490LV and SEM 515.

For the modeling we used the WIEN2k package – a set of computer programs fulfilling the quantum and mechanical calculations in the ranges of density functional theory by means of linearized affiliate plane waves. In all calculations the energy of separation of the electron stations on the valent stations and on main stations was 7 Ry. The quantity of k-points in the Brillouin zone was 100 on the cell for all of the calculations. The following values of nuclear sphere radii were used (for the K-phase elemental cell, *a*_0_ = 0.375 nm): Al = 2.34 a.u., Fe = 1.87 a.u., C = 1.66 a.u. The radii for the other parameters of elemental cells “*a”* were calculated from the ratio *a*/*a*_0_.

## Results and Discussion

According to X-ray analysis of samples synthesized at high pressures and temperatures, in the diffraction patterns both before and after cooling to the temperature of liquid nitrogen, only reflexes of K-phase (with the usual average parameter of an elemental cell, *a* = 0.376 nm), carbide Fe_3_C and cubic diamond were found to be present. Further cooling to the temperature of liquid nitrogen does not lead to new reflexes arising, which testifies to the almost total absence of the metal matrix volumes with a structure dissimilar to K-phase.

Therefore, for the chemical contents and regimes of synthesis of the alloys which were investigated in this work, formation of the phases which could have phase transformations was not possible.

**Fig. 1:**
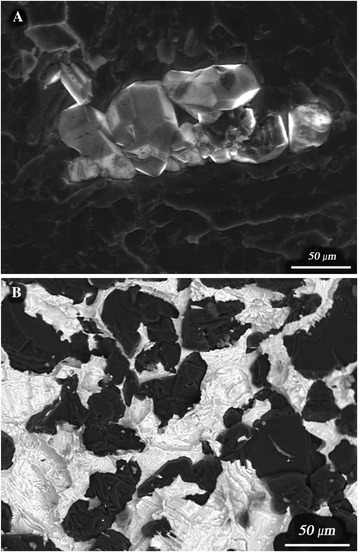
Diamond crystals in the alloys 4 (**a**) and 1 (**b**)

The simultaneous effect of temperature and pressure significantly influences the structural-and-phase state of Fe–Al–C alloys. It has been established that increasing the synthesis temperature to 1673–2173 K under a pressure of 6–8 GPa makes it impossible to achieve complete carbonic stoichiometry of Fe_4–у_Al_у_C_x_ (K-phase) compounds, such as Fe_3_AlC, because they do not increase their solubility in this ordered phase. However, the simultaneous effect of temperature and pressure on Fe–Al–C alloys allows the formation of a new phase – cubic diamond – to take place, at significantly lower temperature and pressure values relative to the standard regimes for other alloys (P = 13 GPa, T = 2273 K. The alloy composition changes resulted only in quantity and form of diamond crystals (Fig. 1).

**Fig. 2:**
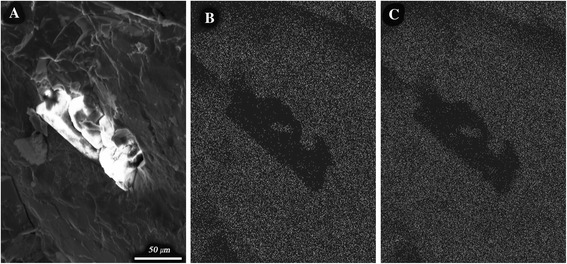
Diamond crystals of three alloys (**a**) and distribution maps of aluminum (**b**) and iron (**c**)

In Fig. 2 the distribution maps of aluminum and iron, obtained from the characteristic irradiation of energy-dispersive spectrometry by the LINK system, are presented. The quantities of aluminum and iron are present in a 1:3 ratio. However, in the diamond crystals there is less aluminum and iron than in the K-phase volume. When the amount of graphite in the source powder mixture is more than 3.9 wt. % the amount of synthetic diamond crystals is significantly increased.

Electron microscopy investigation of synthesized specimens has shown that in Fe–Al–C alloys a quantity of synthetic diamonds of about 20–100 μm was formed and that the large diamond crystals are not perfectly shaped.

As stated by Mikhajlova et al. [11], from all causes of origin of the structure features in Fe–Al–C alloys quenched from high temperatures, the nanogroups having short-range order as K-phase type plays the most significant role. These nanogroups have been found in the liquid state at 1550 °C. Because in different systems the carbide-forming reaction precedes the polymorphic transformation of carbon [9], it seems quite likely that when iron, aluminum, and carbon are fully linked into an ordered structure of Fe_3_AlC_x_ and practically the whole volume of the alloy is K-phase with a f.c.c. crystal lattice, which is the case with the alloys being investigated, the presence of this K-phase is apparently the stimulator [9] of cubic diamond formation from the excess carbon, on a par with iron and aluminum which are contained in the volume of diamond crystals. The ordered Fe_4–у_Al_у_C_x_ phase with its f.c.c. crystal lattice, which surrounds the excess carbon, stimulates cubic diamond formation, thus reducing the required temperature and pressure of diamond formation to 1673 K and 6 GPa, respectively.

By means of modeling of the atomic structure and by calculating the electronic structure it has been established that the energetic expediency of formation of the ordered phase of nonstoichiometric Fe_4–у_Al_у_C_x_ (*y* = 0.5 ÷ 1; *x* = 0.5 ÷ 0.66) compares to the stoichiometric Fe_3_AlC phase. It was also shown that the minimal creating enthalpy value corresponds to the compound Fe_3_AlC_0.66_. The electronic structure investigations have shown that such compounds have the largest binding energy, thereby strengthening the covalent component of the interatomic relation of iron and carbon atoms. Besides, as in all compounds which are nonstoichiometric by carbon, the degenerate states on the Fermi level are missing in the Fe_3_AlC_0.66_ phase, that is the source of possible deformations. Any deviation from the chemical content of Fe_3_AlC_0.66_ on the side of carbonic stoichiometry leads to an essential rise of the enthalpy value. Nonstoichiometry of aluminum conversely leads to weakening of the connections between iron and carbon atoms and, as a result, leads to a rise in enthalpy. Calculation of the cohesion energy of the corresponding compounds confirmed the conclusions obtained for the calculation of enthalpy creation.

Calculations were fulfilled for the following cells: Fe_3_AlC_0.5_, Fe_3_AlC_0.66_, Fe_3.5_Al_0.5_C_0.5_, and Fe_3.5_Al_0.5_C. The optimal parameters of the elemental cells obtained are presented in Table 2. In the second column are presented the optimal parameters of the elemental cells; in the third column are the creation enthalpies corresponding to these parameters; and in the fourth column, for comparison, creation enthalpies of the same cells using the cell parameter 3.75 nm (optimal parameter of the Fe_3_AlC cell) are presented.

**Table 2:** Optimal parameters of elemental cells and values of creation enthalpies

Compound	*a*, nm	Enthalpy of Creation, *eV*/atom	Enthalpy of Creation for *a* = 0, 375 nm
Fe_3_AlC_0.66_	~0, 3725	–0.0348	–0.0328
Fe_3_AlC_0.5_	~0, 372	–0.0335	–0.033
Fe_3_AlC	0, 375	–0.0318	–0.0318
Fe_3.5_Al_0.5_C_0.5_	0, 372	–0.024	–0.0244
Fe_3.5_Al_0.5_C (I)	0, 373	–0.028	–0.0286
Fe_3.5_Al_0.5_C (II)	0, 3735	–0.028	–0.0275

For nonstoichiometric compounds a decrease in elemental cell parameter of 0.8–1% is characterized and the most possible result is the formation of an Fe_3_AlC_0.66_ cell instead of an Fe_3_AlC_0.5_ cell (creation enthalpy, *eV*/atom (–0.0348) and (–0.0335), respectively), as was previously thought. The elemental cell of stoichiometric Fe_3_AlC K-phase has higher creation enthalpy and, therefore, less probability of its creation. This fact confirms the hypothesis of inaccessibility of reaching the stoichiometric content by K-phase.

The difference between the parameters of the elemental cells of K-phase (*a* ≈ 3.75 nm) and diamond (3.57 nm) is 5%. Therefore, it is possible to assume that on the surface, or in the volume, of K-phase the epitaxial growth of diamond crystals is possible. Increasing the pressure under thermal-and-pressure treatment reduces this difference of cell parameters (*a* ≈ 3.69 nm at 7 GPa). The plane of epitaxial growth has the same indexes for all of the investigated cells and the same content of carbon. The only difference is in the environ of iron and aluminum atoms. Fe–octahedron, which is environed by carbon atoms and vacancies, will be constant. This is the [200] plane of K-phase. The plane of epitaxial growth was chosen on the basis of the distances between carbon atoms (or vacancies in their places) having the best accordance to the parameter of the diamond elemental cell. In conclusion, it is possible to make an analogy between epitaxial growth and twinning and martensitic transformations [3, 12, 13].

## Conclusions

In Fe–Al–C alloys after thermal-and-pressure synthesis, the diamond phase with 20–100-μm crystals forms. Thermal-and-pressure influence does not increase the carbon solubility in K-phase by more than 0.66% on the elemental cell of the crystal lattice. The f.c.c lattice of K-phase, which is surrounded by undissolved excess carbon, stimulates the formation of cubic diamond which requires a lower temperature (to 1400 °C) and pressure (to 6GPa) for its formation.

Any deviation from the chemical composition of Fe_3_AlC_0.66_ on the side of carbonic stoichiometry leads to an essential increasing of enthalpy, so the most probable outcome is the formation of Fe_3_AlC_0.66_ cells instead of Fe_3_AlC or Fe_3_AlC_0.5_ cells.

It can be argued that in the substrate of diamond growth there must be a minimum of three to four layers of elemental K-phase cells. Cells with fewer layers require more heat for their formation and are unstable.

Nanoparticles of the ordered Fe_4–у_Al_у_C_x_ phase with a f.c.c. crystal lattice, which are surrounding by excess carbon, stimulate the formation of the cubic diamond, lowering the required temperature and pressure for its formation to 1673 K and 6 GPa, respectively.

## Abbreviations

f.c.c.: Face-centered cubic
